# Self-Serving Episodic Memory Biases: Findings in the Repressive Coping Style

**DOI:** 10.3389/fnbeh.2013.00117

**Published:** 2013-09-03

**Authors:** Lauren L. Alston, Carissa Kratchmer, Anna Jeznach, Nathan T. Bartlett, Patrick S. R. Davidson, Esther Fujiwara

**Affiliations:** ^1^Centre for Neuroscience, University of Alberta, Edmonton, AB, Canada; ^2^Department of Psychiatry, University of Alberta, Edmonton, AB, Canada; ^3^Department of Psychology, University of Victoria, Victoria, BC, Canada; ^4^School of Psychology, University of Ottawa, Ottawa, ON, Canada

**Keywords:** memory, repressive coping style, self-relevance, delay, valence

## Abstract

Individuals with a repressive coping style self-report low anxiety, but show high defensiveness and high physiological arousal. Repressors have impoverished negative autobiographical memories and are better able to suppress memory for negatively valenced and self-related laboratory materials when asked to do so. Research on spontaneous forgetting of negative information in repressors suggests that they show significant forgetting of negative items, but only after a delay. Unknown is whether increased forgetting after a delay is potentiated by self-relevance. Here we asked in three experiments whether repressors would show reduced episodic memories for negative self-relevant information when tested immediately versus after a 2-day delay. We predicted that repressors would show an exaggerated reduction in recall of negative self-relevant memories after a delay, at least without anew priming of this information. We tested a total of 300 participants (experiment 1: *N* = 95, experiment 2: *N* = 106; experiment 3: *N* = 99) of four types: repressors, high-anxious (HA), low-anxious, and defensive HA individuals. Participants judged positive and negative adjectives with regard to self-descriptiveness, serving as incidental encoding. Surprise free-recall was conducted immediately after encoding (experiment 1), after a 2-day delay (experiment 2), or after a 2-day delay following priming via a lexical decision task (experiment 3). In experiment 1, repressors showed a bias against negative self-relevant words in immediate recall. Such a bias was neither observed in delayed recall without priming nor in delayed recall with priming. Thus, counter to our hypothesis, negative information that was initially judged as self-relevant was not forgotten at a higher rate after a delay in repressors. We suggest that repressors may reinterpret initially negative information in a more positive light after a delay, and therefore no longer experience the need to bias their recall after a delay.

## Introduction

Repression is a putative psychological defense mechanism (Freud, 1957/1915) that inhibits anxiety-provoking, ego-threatening thoughts from entering consciousness. Debated for over a century, there has been little empirical evidence for the existence of repression, either as a trauma-specific or a general mechanism of forgetting (Erdelyi, 2006). An alternative is to measure differences between individuals’ repressive tendencies. The most studied approach by Weinberger et al. (1979) defines “repressors” as individuals who score high on self-report measures of defensiveness and low in measures of trait-anxiety. Thus, scores in two questionnaires are combined, trait-anxiety and trait-defensiveness. As a consequence, three additional groups of individuals are identified: “Low-anxious (LA)” individuals score low in trait-anxiety and low in trait-defensiveness. These are thought to be people with “truly” low anxiety since they do not respond defensively in questionnaires, unlike repressors. “High-anxious (HA)” individuals score low in defensiveness but high in trait-anxiety; and “defensive HA” individuals have high scores on both scales. A crucial finding is that repressors exhibit physiologically high levels of anxiety-related arousal despite their low self-reported anxiety (Weinberger et al., 1979; Broomfield and Turpin, 2005). Thus, by this operational definition, repressors have high levels of unacknowledged anxiety. Importantly, repressive coping style has been associated with deleterious effects on physical well-being, most consistently an increased risk for hypertension, cardiovascular diseases, and cancer (Mund and Mitte, 2012).

The motivation to maintain a positive self-view and avoid the experience of anxiety may drive repressors more than others to truncate, inhibit, or otherwise alter negative information processing. Indeed, repressors show biases against processing negative information. For example, compared to non-repressors, repressors recall fewer and less detailed negative autobiographical memories (e.g., Davis and Schwartz, 1987; Davis, 1990; Dickson et al., 2009; Geraerts et al., 2012). When directly instructed to inhibit processing of negative information, repressors are superior to non-repressors in (negative) thought suppression (Barnier et al., 2004; Geraerts et al., 2006, 2007). Repressors’ superior inhibition of negative information has also been shown in memory. For example, repressors had increased forgetting of negative self-related materials in list-method directed forgetting paradigms (Myers et al., 1998; Myers and Derakshan, 2004). They were also better able to perform the think/no-think task (Anderson and Green, 2001) than non-repressors, at least when they were given positive target memories to recall instead of negative ones (Hertel and McDaniel, 2010). Thus, active inhibition of negative, and especially, self-relevant memories may play a role in repressors’ selective forgetting of information in experimental contexts in which they are told to forget.

Repressors’ naturally occurring, uninstructed forgetting of laboratory materials has been more variable. Brosschot et al. (1997) found nothing remarkable about repressors’ free-recall and recognition of unpleasant (and pleasant) words. Similarly, Oldenburg et al. (2002) found neither implicit nor explicit memory biases against negative materials in repressors. Avero et al. (2003) found that individuals with an avoidant coping style (similar to the repressive coping style) had a more conservative response bias (β) but intact recognition sensitivity (d-prime) in recognition memory for negative words than other people. That is, repressors compared to non-repressors judged negative words (both target words and lure words) less often as “old items” in a recognition memory test, even though their ability to differentiate successfully between negative target words and negative lure words was intact. Focussing on self-related information rather than just valenced materials, Saunders et al. (2012) found in a series of studies that repressors are most susceptible to the mnemic neglect effect (Green and Sedikides, 2004; Green et al., 2008) compared to individuals with other coping styles. In mnemic neglect studies, participants are exposed to hypothetical scenarios that involve either themselves or a different person. These scenarios imply favorable or unfavorable personality traits (e.g., “I would keep secrets when asked to”) with different importance to people’s self-concept and different modifiability. In general, a “mnemic neglect” is reflected in reduced recall of situations that involve the self, and unfavorable, personally important, and unmodifiable characteristics. Repressors in Saunders et al. (2012) showed the strongest such mnemic neglect effects in a series of three experiments. Thus, personal involvement or significance, in addition to negative valence might be important to evoke spontaneous, i.e., unprovoked, memory biases in repressive individuals.

Hock, Krohne, and colleagues (Hock and Krohne, 2004; Krohne and Hock, 2008, 2011; Peters et al., 2012) define and measure repressive coping style using the Mainz Coping Inventory (MCI: Krohne and Egloff, 1989; Krohne et al., 2000). The MCI does not rely on the combination of defensiveness and anxiety but incorporates individual differences in information processing styles. MCI-repressors are thought to be motivated to control their experience of anxiety-related arousal and characterized by high dispositional threat avoidance. Their most extreme counterpart, sensitizers, are thought to be motivated to control potential dangers resulting from fearful situations. Therefore, MCI-based sensitizers are thought to be uncertainty-oriented and show high dispositional threat vigilance. Using this operationalization, repressors were found to show no biases against threat-related information in immediate retrieval (recognition memory for negative words/sentences or pictures), but those biases emerged only when testing was delayed by three days (Hock and Krohne, 2004; Krohne and Hock, 2008; see also Peters et al., 2012). This pattern has also been termed *repressive discontinuity hypothesis* indicating that repressors may have an early attentional bias toward threat but selectively retain non-threat and forget threat-information later on (Calvo and Eysenck, 2000; Caldwell and Newman, 2005; Derakshan et al., 2007; Paul et al., 2012). Krohne and Hock (2011) suggested based on the schema pointer plus tag model of Graesser and Bower (1990) that retrieval may co-vary with the level of atypicality of memories. According to this model, schema-incongruent information is better recalled at immediate testing and schema-congruent information is better recalled at delayed testing. If repressors have fewer threat-schemata than non-repressors, they should effectively retrieve threat-information immediately, but have reduced recall of (schema-incongruent) threat-related information at a delayed test. This is consistent with their findings (Hock and Krohne, 2004; Krohne and Hock, 2008; Peters et al., 2012).

If the main motivation of a repressive coping style is self-protection, one would expect to see decreased memory predominantly in tasks that require relating negative thoughts and memories to oneself (Myers and Derakshan, 2004; Saunders et al., 2012). Consequently, it seems likely that differences in personal involvement evoked by particular tasks may have contributed to the variability of the aforementioned results. For instance, the null findings of Brosschot et al. (1997) may have been due to participants simply rating stimuli for pleasantness and threat, a task that can be performed with minimal reference to oneself. Along this line, Fujiwara et al. (2008) also found no alterations in repressors’ recall or priming for negative information they had judged for valence. In contrast, within the same study, self-descriptiveness judgments critically mediated forgetting in repressors: non-repressors had better free-recall of negative self-descriptive words compared to negative non-self-descriptive words. In contrast, repressors showed no such free-recall benefit for negative self-descriptive information. Despite these group differences in free-recall, implicit memory (priming) for negative self-descriptive information was similar across groups. Thus, self-descriptiveness mediated free-recall (but not priming) biases against negative information even at an immediate test in our previous study. However, unknown is whether such self-serving rather than just valence-driven bias would become stronger over time.

Thus, we compared here whether repressive coping style mediated free-recall of words previously judged as self-descriptive at an immediate test (conceptual replication of Fujiwara et al., 2008), and at a delayed test. First, we expected to replicate our previous result and predicted that all groups but repressors should show a recall advantage of negative self-descriptive over negative information that was not self-descriptive in immediate test (experiment 1). Secondly, we expected that these immediate biases would become more pronounced with a delay (experiment 2). Thus, if schema-congruency drives delayed retrieval more so than immediate retrieval and repressors possess fewer negative self-schemata than non-repressors, repressors may retrieve even less negative self-descriptive information after a delay than immediately. Finally, we tested whether potential recall biases at delayed testing could be influenced by cueing all original materials prior to free-recall using a lexical decision task (experiment 3). Based on autobiographical memory work by Davis and others (Davis and Schwartz, 1987; Davis, 1990), who showed that repressors can be cued to retrieve negative autobiographical information that they did not report otherwise, we expected that repressors may not show conceivable biases against recalling negative self-descriptive information if they had been cued immediately prior to free-recall at the delayed test. We did not expect repressor-specific alterations of implicit memory in the lexical decision task after the delay. As we reported previously (Fujiwara et al., 2008), priming for negative self-relevant information had been intact in repressors at immediate test, and we did not expect this to change after a delay.

## Materials and Methods

### Participants

Participants were a total of 351 introductory psychology students at the University of Alberta. Participants gave written informed consent prior to the study, which was approved by a University of Alberta Research Ethics Board. In online mass-testing sessions at the beginning of fall and winter semesters between 2007 and 2010, all students enrolled in an introductory psychology course (between 1500 and 2500 students in each fall/winter term) completed the Trait-version of the State-Trait-Anxiety Inventory (STAI-T; Spielberger et al., 1983) and the Self-Deceptive Enhancement (SDE) component of the Balanced Inventory of Desirable Responding scale (BIDR; Paulhus, 1991), in this fixed order. The STAI-T consists of 24-point scaled statements measuring trait-anxiety (maximum score: 80). The BIDR-SDE consists of 27-point scaled statements that measure self-deceptive aspects of social desirability, such as beliefs of invincibility and exaggerated optimism (maximum score: 140). Only native English speakers with complete questionnaire and demographic data as well as below 30 years of age were included. Participants were categorized into four coping styles according to Weinberger’s classification scheme (Weinberger et al., 1979) based on quartile splits of BIDR-SDE scores (cut-off: 74 and 92 points, for lowest and highest quartile respectively) and median splits on STAI-T scores (43 points) of the 1539 eligible students tested in the 2007 fall semester. Subsequent semesters used the same cut-off scores to ensure consistency across samples. Participants in each semester were classified as repressors (REP: low-anxious, high-defensive), truly low-anxious (LA: low-anxious, low-defensive), truly high-anxious (HA: high-anxious, low-defensive), and defensive high-anxious (DHA: high-anxious, high-defensive). Based on the BIDR-SDE and STAI-T cut-off scores, a first enrolment wave in each semester allowed equally sized groups of participants with one of the four coping styles online access to self-enroll in the experiments. The size of the groups that received access to the experiment was determined by the maximum number of participants in the smallest of the four groups (usually, the DHA). Additional enrolment waves were initiated when participation rates started to decline usually around midway through the semesters, giving more participants access to the experiment. These participants were usually more REP and HA as these two groups were more common than LA and especially than DHA. Students were not aware of the nature of the experiment at the time of self-enrolment and testers were not aware of the participants’ coping style at the time of the experiment. In experiment 1, 99 students participated, 122 students participated in experiment 2, and 130 in experiment 3. Data from 52 participants were excluded: 22 had partial data or otherwise did not comply with the task instructions (e.g., less than 50% valid trials, less than two words in free-recall), 19 did not return after the delay to complete the experiment, 3 experienced a computer error, and 8 participants’ pre-selection questionnaire data were erroneous.

The final sample in experiment 1 included 95 participants. A total of 22 (15 female) LA, 26 (13 female) HA, 28 (14 female) REP, and 19 (11 female) DHA individuals participated, with an average age of 20.02 ± 1.77 years. Gender was equally distributed across groups [χ^2^(3) = 2.14, *p* > 0.1]. The final sample in experiment 2 included 106 participants. A total of 26 (21 female) LA, 26 (16 female) HA, 31 (14 female) REP, and 23 (14 female) DHA individuals participated, with an average age of 19.00 ± 1.66 years. Although there were slightly more female participants in the LA group than in the other groups, the gender distribution was not statistically different across all groups [χ^2^(3) = 7.56, *p* > 0.05]. Finally, experiment 3 included 99 participants. A total of 27 (16 female) LA, 28 (16 female) HA, 23 (10 female) REP, and 21 (14 female) DHA individuals participated, with an average age of 18.86 ± 1.93 years. Gender was equally distributed across groups [χ^2^(3) = 2.56, *p* > 0.1]. Participants’ questionnaire data are summarized in Table 1.

**Table 1:** **Means (*M*) and standard deviations (SD) of questionnaire data across groups in all three experiments**.

	LA		HA		REP		DHA	
	*M*	SD	*M*	SD	*M*	SD	*M*	SD
**EXPERIMENT 1**								
STAI-T	36.00^a^	2.88	53.00^b^	7.52	35.14^a^	3.58	51.47^b^	6.50
BIDR-SDE	69.90^c^	3.94	68.65^c^	4.76	99.04^d^	5.20	96.42^d^	4.85
**EXPERIMENT 2**								
STAI-T	38.88^a^	3.51	50.92^b^	6.51	37.35^a^	2.20	48.13^b^	4.78
BIDR-SDE	70.87^c^	4.12	69.34^c^	8.09	96.42^d^	5.30	94.09^d^	3.06
**EXPERIMENT 3**								
STAI-T	37.85^a^	3.03	48.82^b^	3.57	35.13^a^	2.69	48.05^b^	5.12
BIDR-SDE	70.85^c^	3.31	68.86^c^	4.45	98.82^d^	7.06	95.19^d^	4.24

*STAI-T, trait-version of the State-Trait-Anxiety Inventory (Spielberger et al., 1983); BIDR-SDE, self-deceptive enhancement scale of the Balanced Inventory of Desirable Responding (Paulhus, 1991); LA, low-anxious; HA, high-anxious; REP, repressor; DHA, defensive high-anxious. Cells with different superscripts show significant between-group differences within each experiment, indicated by *post hoc* Scheffé test (p < 0.001)*.

As intended, in each experiment, anxiety (STAI-T) was significantly different across groups [experiment 1: *F*(3, 91) = 75.97; experiment 2: *F*(3, 102) = 61.54; experiment 3: *F*(3, 95) = 89.91, all *p*’s < 0.001], and so was defensiveness (BIDR-SDE) [experiment 1: *F*(3, 91) = 292.81; experiment 2: *F*(3, 104) = 187; experiment 3: *F*(3, 95) = 255.26, all *p*’s < 0.001]. *Post hoc* Scheffé tests indicated higher anxiety in HA/DHA than in LA/REP groups, but no differences between groups with high anxiety scores (HA, DHA), or between groups with low anxiety scores (LA, REP). Likewise, defensiveness was always substantially higher in groups intended to have high defensiveness (REP, DHA) than in those with low-defensiveness (LA, HA), but never differed between REP and DHA or between LA and HA (all *p*’s < 0.001).

### Materials and tasks

Stimuli were personality trait words drawn from Anderson (1968). Based on median splits of likeableness, we created two matched sets, each with 75 likeable and 75 non-likeable words (hereafter termed “positive” and “negative,” respectively). Sets were equated in word length (3–10 letters), statistical frequency, and meaningfulness (for details see Fujiwara et al., 2008). Half the participants in each experiment received set 1 or set 2 during the encoding task. The unused words (i.e., either set 1 or set 2, depending on its use in the encoding task) were included in experiment 3 as new words in the lexical decision task. The fixed task order was as follows: experiment 1: self-referential judgments, free-recall. Experiment 2: self-referential judgments, 2-day delay, free-recall. Experiment 3: self-referential judgments, 2-day delay, lexical decision task, free-recall.

#### Encoding task

Participants were presented with 150 personality trait words (75 positive, 75 negative). Participants were asked to judge how descriptive each word was of themselves, on a four-point scale: “1” = very self-descriptive, “2” = moderately self-descriptive, “3” = moderately not-self-descriptive, and “4” = not at all self-descriptive. In each trial, a fixation cross appeared for 500 ms, followed by a centrally presented word (3000 ms) at which time participants would make their self-descriptiveness rating. Six practice trials were given. Eight filler words were presented at the end of the task to avoid recency effects. Practice and filler words were excluded from the analyses. Dependent variables were the proportions of word judgments, summarized into composite measures described in more detail in Section “Statistical Analyses.”

#### Free-recall

Participants were given 5 minutes to write on a piece of paper in any order as many words as they could recall from the encoding task. The dependent variable was the proportion of words recalled.

#### Lexical decision task (experiment 3 only)

Participants were presented the 150 words they had judged in the encoding task, 150 new words (either word set 1 or set 2, see above), as well as 300 pronounceable non-word letter strings. Participants were asked to judge each letter string as a word or a non-word. Speed and accuracy were emphasized equally. Practice trials contained six words and three non-words. None of them were included in the experiment. Each trial had the following sequence: a fixation cross would appear for 1000 ms followed by a 150-ms presentation of the letter string, after which the participant had to respond (identical to Fujiwara et al., 2008). The dependent variable was response time.

Except for free-recall, tasks were administered using Presentation software (www.neurobs.com) on laptop computers. Stimuli were presented in light gray, 36-point Arial font in the center of a black screen. All three experiments were conducted in small groups of one to three participants at a time. Participants were facing opposite walls to prevent them from watching each other perform the tasks.

#### Statistical analyses

To avoid empty cells, self-descriptiveness judgments were collapsed from four into two response categories. The judgments “very self-descriptive” and “moderately self-descriptive” were coded together as “self-descriptive”; “moderately not-self-descriptive” and “not at all self-descriptive” judgments were coded as “not-self-descriptive.”

As self-referential judgments are mutually exclusive (each word can be judged as either self-descriptive or not, but not both), we derived two composite measures to illustrate performance in the encoding task. First, we calculated a “self-judgment ratio” by dividing the proportion of all words judged as self-descriptive by proportions of words judged as not-self-descriptive, and subsequently log-transforming this ratio to make the distribution symmetric. Positive scores indicate more self-descriptive than not-self-descriptive judgments, regardless of valence. Secondly, we calculated a “positivity-judgment ratio” by dividing the proportion of favorable judgments (negative not-self-descriptive judgments and positive self-descriptive judgments) by the proportion of unfavorable judgments (negative self-descriptive judgments and positive not-self-descriptive judgments), and log-transforming this ratio. Positive scores indicate more favorable self-judgments than unfavorable self-judgments. The two ratios were compared with univariate ANOVA and between-subject factors anxiety (low/high) and defensiveness (low/high), separately for all three experiments. Splitting up the two components comprising the coping styles (anxiety, defensiveness) allowed us to investigate whether the interaction of anxiety and defensiveness (i.e., the repressive coping style) influenced our results over and above anxiety or defensiveness alone^1^. Free-recall and priming effects were analyzed with mixed repeated-measures ANOVA separately for each of the three experiments, consistent with our previous study (Fujiwara et al., 2008). Performance was calculated as proportions of words identified in priming or recalled in free-recall relative to each individual’s total number of negative and positive self-descriptive or not-self-descriptive words from the prior judgment task (possible maximum of 75 negative and 75 positive words). Thus, the dependent variables: proportional recall and priming were analyzed as a function of within-subject factors valence (positive/negative) and prior self-judgment from the encoding task (self-descriptive/not-self-descriptive). Between-subject factors were anxiety (low/high) and defensiveness (low/high).

To control for possible influences of individual compared to small group testing conditions, we included presence/absence of other participants at encoding or free-recall as a dummy-coded categorical covariate in all analyses.

Responses faster than 200 ms or three standard deviations above or below an individual’s mean response time were excluded from all analyses. In the lexical decision task, priming was measured as the amount of time required to identify new words minus the time required to identify old words. Collapsed across factor levels, all dependent variables were normally distributed, assessed with Kolmogorov–Smirnov tests. Our statistical significance level was set to *p* < 0.05.

## Results

### Encoding task

Table 2 gives an overview on the encoding task results across all three experiments.

**Table 2:** **Means (*M*) and standard deviations (SD) of judgment proportions (out of 150 words) in the encoding task**.

	LA		HA		REP		DHA		Total	
	*M*	SD	*M*	SD	*M*	SD	*M*	SD	*M*	SD
**EXPERIMENT 1**										
Self	0.49	0.08	0.51	0.06	0.51	0.06	0.53	0.06	0.51	0.07
Not-self	0.47	0.08	0.44	0.07	0.45	0.06	0.44	0.06	0.45	0.07
Self-negativity	0.32	0.13	0.27	0.11	0.19	0.07	0.26	0.10	0.26	0.11
Self-positivity	0.63	0.14	0.69	0.10	0.77	0.07	0.71	0.10	0.71	0.12
Self-judgment ratio	0.06	0.35	0.14	0.29	0.12	0.23	0.17	0.24	0.12	0.28
Self-positivity ratio	0.72	0.68	1.02	0.55	1.45	0.45	1.08	0.58	1.09	0.12
**EXPERIMENT 2**										
Self	0.48	0.07	0.52	0.09	0.49	0.06	0.52	0.07	0.50	0.08
Not-self	0.48	0.08	0.44	0.10	0.48	0.06	0.46	0.09	0.47	0.08
Self-negativity	0.27	0.05	0.33	0.12	0.20	0.09	0.30	0.12	0.27	0.11
Self-positivity	0.69	0.08	0.63	0.12	0.76	0.08	0.67	0.12	0.69	0.11
Self-judgment ratio	−0.01	0.32	0.18	0.42	0.02	0.27	0.13	0.34	0.07	0.34
Self-positivity ratio	0.99	0.42	0.68	0.56	1.41	0.55	0.83	0.58	1.01	0.60
**EXPERIMENT 3**										
Self	0.49	0.07	0.52	0.08	0.50	0.08	0.50	0.08	0.51	0.08
Not-self	0.47	0.07	0.45	0.09	0.46	0.09	0.46	0.09	0.46	0.08
Self-negativity	0.29	0.11	0.28	0.09	0.20	0.10	0.25	0.08	0.26	0.10
Self-positivity	0.67	0.12	0.69	0.09	0.76	0.11	0.71	0.09	0.71	0.11
Self-judgment ratio	0.06	0.31	0.16	0.35	0.10	0.40	0.09	0.35	0.10	0.35
Self-positivity ratio	0.87	0.60	0.95	0.47	1.42	0.63	1.10	0.48	1.07	0.58

*Self: self-descriptive judgments; Not-self: not-self-descriptive judgments; Self-positivity: positive self-descriptive and negative not-self-descriptive judgments; Self-negativity: positive not-self-descriptive and negative self-descriptive judgments; Self-judgment ratio: log-transformed self/not-self; Self-positivity ratio: log-transformed self-positivity/self-negativity. LA, low-anxious; HA, high-anxious; REP, repressor; DHA, defensive high-anxious*.

As can be seen in Table 2, participants on average judged slightly more than 50% of all words as self-descriptive and about 70% of their judgments were favorable. The self-judgment ratio was above zero in all experiments [experiment 1: *t*(94) = 4.2, *p* < 0.001, Cohen’s *d* = 0.9; experiment 2: *t*(105) = 2.01, *p* < 0.05, *d* = 0.4; experiment 3: *t*(98) = 2.95, *p* < 0.01, *d* = 0.6], indicating more self-descriptive than not-self-descriptive judgments. Controlling for presence/absence of other participants during the experiment [experiment 1: *F*(1, 90) = 0.003, *p* > 0.1, η_partial_^2^ < 0.0001; experiment 2: *F*(1, 101) = 0.74, *p* > 0.1, η_partial_^2^ = 0.007; experiment 3: *F*(1, 94) = 0.14, *p* > 0.1, η_partial_^2^ = 0.001], this ratio was not influenced by groupings of anxiety, defensiveness, or their interaction in experiments 1 and 3 (all *p*’s > 0.1; all η_partial_^2^ < 0.02). However, anxiety grouping did show a main effect in experiment 2 [*F*(1, 101) = 7.38, *p* < 0.01, η_partial_^2^ = 0.07] indicating more self-judgments than not-self-judgments in higher anxious groups. There was no main effect of defensiveness [*F*(1, 101) = 0.08, *p* > . 1, η_partial_^2^ = 0.001] on the self-judgment ratio and neither an interaction between anxiety and defensiveness [*F*(1, 101) = 0.30, *p* > 0.1, η_partial_^2^ < 0.003]. Thus, all participants tended to make more self-descriptive than not-self-descriptive judgments in all experiments, although this tendency was attenuated in individuals with lower anxiety in experiment 2.

The positivity-judgment ratio was substantially above zero in all experiments [experiment 1: *t*(94) = 17.21, *p* < 0.001, *d* = 3.55; experiment 2: *t*(105) = 17.49, *p* < 0.001, *d* = 3.41; experiment 3: *t*(98) = 18.37, *p* < 0.001, *d* = 3.71], indicating more favorable than unfavorable judgments in general. Controlling for presence/absence of other participants during the experiment [experiment 1: *F*(1, 90) = 1.64, *p* > 0.1, η_partial_^2^ = 0.02; experiment 2: *F*(1, 101) = 0.18, *p* > 0.1, η_partial_^2^ = 0.002; experiment 3: *F*(1, 94) = 0.96, *p* > 0.1, η_partial_^2^ = 0.01], the positivity-judgment ratio differed significantly across groups. Defensiveness showed main effects in all three experiments [experiment 1: *F*(1, 90) = 11.9, *p* < 0.001, η_partial_^2^ = 0.12; experiment 2: *F*(1, 101) = 8.15, *p* < 0.01, η_partial_^2^ = 0.08; experiment 3: *F*(1, 94) = 10.29, *p* < 0.01, η_partial_^2^ = 0.1], indicating more self-positive judgments in individuals with high defensiveness than in low-defensive individuals. In addition, anxiety showed a main effect in experiment 2 only [experiment 2: *F*(1, 101) = 20.0, *p* < 0.001, η_partial_^2^ = 0.17] with a less pronounced positivity-judgment ratio in high compared to low anxiety groups. A significant interaction between anxiety and defensiveness grouping was observed in experiment 1 [*F*(1, 90) = 7.53, *p* < 0.01, η_partial_^2^ = 0.08]. Following up on this interaction, comparing all four separate groups with a one-way ANOVA [*F*(3, 91) = 7.2, *p* < 0.001] and *post hoc* Scheffé tests showed a significantly higher positivity-judgment ratio in the REP group than in the LA group. While present in similar but weaker form, the interaction did not reach significance in experiment 2 [*F*(1, 101) = 2.05, *p* > 0.1, η_partial_^2^ = 0.02] or experiment 3 [*F*(1, 94) = 3.18, *p* < 0.1, η_partial_^2^ = 0.033].

In summary, while all four groups showed a sizable preference to make favorable self-judgments (endorsing positive words as self-descriptive and rejecting negative words as not-self-descriptive), this preference was reliably increased in higher defensive participants across all experiments; and attenuated in individuals with higher anxiety in experiment 2 only. In REP, this tendency had a similar size across experiments and was, numerically, the largest of each of the four groups in all experiments.

### Free-recall

Conditional on the self-descriptiveness judgments from the encoding task, we then analyzed the proportions of words retrieved in free-recall in each of the experiments, as a function of within-subjects factors valence (positive/negative) and prior self-judgment from the encoding task (self-descriptive/not-self-descriptive), and between-subject factors anxiety (low/high) and defensiveness (low/high), again, controlling for presence/absence of other participants during the experiment. Means and standard deviations of proportional free-recall are shown in Table 3.

**Table 3:** **Means (*M*) and standard deviations (SD) of recall proportions of words as they had been judged in encoding**.

	LA		HA		REP		DHA		Total	
	*M*	SD	*M*	SD	*M*	SD	*M*	SD	*M*	SD
**EXPERIMENT 1**										
Negative self	0.16	0.09	0.14	0.10	0.11	0.10	0.17	0.09	0.14	0.10
Negative not-self	0.10	0.05	0.10	0.05	0.10	0.05	0.11	0.05	0.10	0.05
Positive self	0.13	0.05	0.15	0.07	0.13	0.06	0.15	0.06	0.14	0.06
Positive not-self	0.12	0.06	0.09	0.07	0.11	0.11	0.12	0.08	0.11	0.08
**EXPERIMENT 2**										
Negative self	0.09	0.10	0.06	0.06	0.07	0.09	0.09	0.06	0.08	0.08
Negative not-self	0.04	0.03	0.03	0.03	0.04	0.02	0.04	0.03	0.04	0.03
Positive self	0.10	0.05	0.06	0.04	0.08	0.04	0.09	0.07	0.08	0.05
Positive not-self	0.05	0.05	0.04	0.05	0.04	0.06	0.06	0.07	0.05	0.06
**EXPERIMENT 3**										
Negative self	0.08	0.09	0.10	0.06	0.10	0.10	0.06	0.07	0.08	0.08
Negative not-self	0.07	0.04	0.06	0.04	0.05	0.04	0.06	0.03	0.06	0.04
Positive self	0.09	0.05	0.08	0.05	0.09	0.05	0.08	0.05	0.08	0.05
Positive not-self	0.06	0.06	0.08	0.09	0.04	0.06	0.04	0.06	0.06	0.07

*Self: self-descriptive; Not-self: not-self-descriptive. LA, low-anxious; HA, high-anxious; REP, repressor; DHA, defensive high-anxious*.

**Figure 1:**
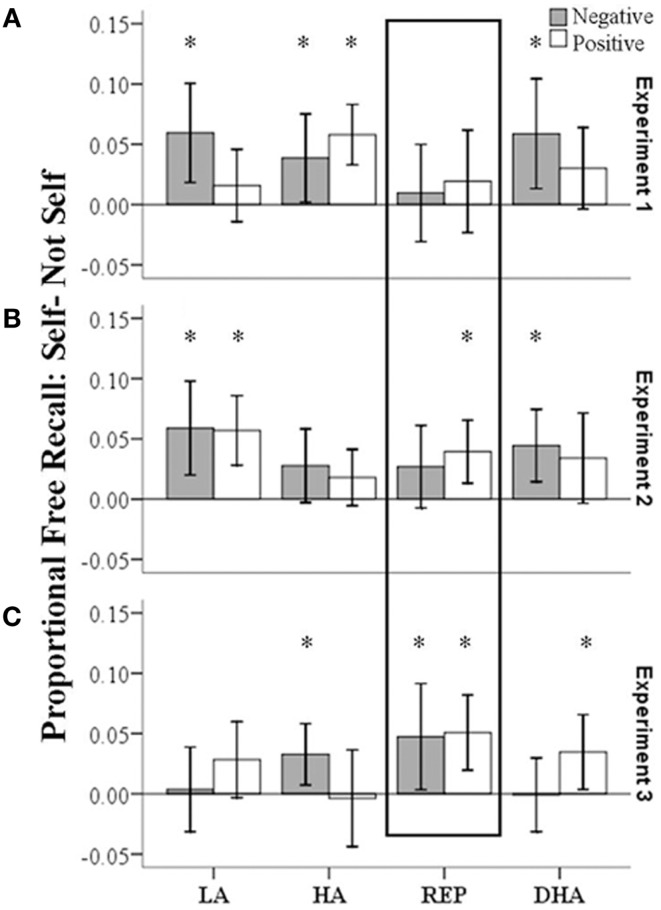
**Differences in recall proportions of words previously judged as self-descriptive minus words judged as not-self-descriptive**. **(A)**: experiment 1, **(B)**: experiment 2, **(C)**: experiment 3. LA, low-anxious; HA, high-anxious; REP, repressor; DHA, defensive high-anxious. Asterisks indicate significantly better recall of self-descriptive compared to not self-descriptive words.

In experiment 1 (immediate recall), controlling for the covariate “presence/absence of other participants during the experiment,” we observed a main effect of self-judgment [*F*(1, 90) = 10.10, *p* < 0.01, η_partial_^2^ = 0.1], indicating better recall of words that were previously judged as self-descriptive than words that were judged as not-self-descriptive. Furthermore, we observed a four-way interaction between valence, self-judgment, anxiety, and defensiveness, just reaching significance [*F*(1, 90) = 4.23, *p* < 0.05, η_partial_^2^ = 0.05]. To follow up on this interaction, we first compared all four recall proportions across groups with four one-way ANOVA. These were not significant [negative self-descriptive: *F*(1, 91) = 1.56, *p* = 0.21; negative not-self-descriptive: *F*(1, 91) = 0.48, *p* = 0.7; positive self-descriptive: *F*(1, 91) = 0.72, *p* = 0.55; positive not-self-descriptive: *F*(1, 91) = 0.43, *p* = 0.73]. Secondly, we conducted mixed repeated-measures ANOVA within each of the four groups. Due to small cell sizes the covariate was omitted for these analyses. We observed main effects of self-judgment in LA [*F*(1, 21) = 9.5, *p* < 0.01, η_partial_^2^ = 0.31], HA [*F*(1, 25) = 17.37, *p* < 0.001, η_partial_^2^ = 0.41], and DHA [*F*(1, 18) = 8.2, *p* < 0.01, η_partial_^2^ = 0.31], indicating better recall of self-descriptive than not-descriptive information. This main effect was not present in REP [*F*(1, 27) = 1.07, *p* = 0.39, η_partial_^2^ = 0.038]. Finally, dependent-samples *t*-tests were conducted within each group (see Figure 1A for an illustration of the free-recall advantages due to self-descriptiveness and valence in each group in experiment 1). We found that within the positive words, only HA individuals had a recall advantage of positive self-descriptive compared to positive not-self-descriptive words [*t*(25) = 4.85, *p* < 0.01, *d* = 1.94]. However, we observed better recall of negative self-descriptive than negative not-self-descriptive words in all groups except the REP group, differences with moderate to large effect sizes [LA: *t*(21) = 3.00, *p* < 0.01, *d* = 1.31; HA: *t*(25) = 2.14, *p* < 0.05, *d* = 0.86; DHA: *t*(18) = 2.66, *p* < 0.05, *d* = 1.25]. The REP group was the only group without a significant free-recall advantage for negative self-descriptive words over negative not-self-descriptive words in immediate recall [*t*(27) = 0.54, *p* > 0.1, *d* = 0.21]. This result conceptually replicates Fujiwara et al. (2008).

Would this differential recall pattern become more pronounced with a delay? To answer this question and address hypothesis 2, free-recall data from experiment 2 (delayed recall) was analyzed in the same way (see Table 3; Figure 1B). Similar as in experiment 1, we observed a main effect of self-judgment [*F*(1, 101) = 21.23, *p* < 0.001, η_partial_^2^ = 0.17], controlling for the covariate “presence/absence of other participants during the experiment.” The main effect indicated better recall of words that were previously judged as self-descriptive compared to words that were judged as not-self-descriptive. Furthermore, we found an interaction between anxiety and defensiveness [*F*(1, 101) = 8.35, *p* < 0.01, η_partial_^2^ = 0.08]. A one-way ANOVA comparing groups on overall recall proportions [*F*(1, 102) = 7.85, *p* < 0.05] and *post hoc* Scheffé tests indicated that LA participants had better overall free-recall than HA participants (LA: mean = 0.07 ± 0.03; HA: mean = 0.05 ± 0.02). No further main effects or interactions were observed. Specifically, there was no significant four-way interaction [*F*(1, 101) = 0.12, *p* > 0.1, η_partial_^2^ = 0.001], contrary to experiment 1. Of note, there were differences in experiments 1 and 2: we had detected anxiety main effects in both of judgment ratios only in experiment 2, pointing to possible anxiety-based differences in numbers of negative and positive items judged as self-descriptive or not-self-descriptive in experiment 2. Thus, a repeated-measures ANCOVA on free-recall proportions in experiment 2 was conducted including both judgment ratios as covariates. The results of this ANCOVA were similar: controlling for the judgment ratios, the four-way interaction was still not significant [*F*(1, 99) = 0.27, *p* > 0.1, η_partial_^2^ = 0.003]. Thus, counter to our second hypothesis, REP had no reduced recall of negative self-descriptive information after the delay compared to any of the other groups and we did not observe an exaggeration of the within-group difference we had observed in experiment 1.

Experiment 3 (delayed recall with priming), which was intended to prime potential recall failures for unfavorable information in REP, is presented next (cf. Table 3; Figure 1C). Controlling for the covariate “presence/absence of other participants during the experiment,” we observed a main effect of self-judgment [*F*(1, 94) = 12.08, *p* < 0.001, η_partial_^2^ = 0.11], indicating better recall of words that were previously judged as self-descriptive than words that were judged as not-self-descriptive. Although this effect was similar to that observed in experiments 1 and 2, the effect size was smaller. The main effect was qualified by a four-way interaction between valence, self-judgment, anxiety, and defensiveness [*F*(1, 94) = 4.97, *p* < 0.05, η_partial_^2^ = 0.05]. Following up on this interaction, there were no between-group differences in any of the four recall proportions [negative self-descriptive: *F*(3, 95) = 1.23, *p* > 0.1; negative not-self-descriptive: *F*(3, 95) = 1.39, *p* > 0.1; positive self-descriptive: *F*(3, 95) = 0.54, *p* > 0.1; positive not-self-descriptive: *F*(3, 95) = 1.89, *p* > 0.1]. Secondly, we conducted the mixed repeated-measures ANOVA within each of the four groups separately. The main effect of self-judgment was only present in REP [*F*(1, 22) = 11.36, *p* < 0.01, η_partial_^2^ = 0.34], but not in LA [*F*(1, 26) = 1.32, *p* > 0.1, η_partial_^2^ = 0.04], HA [*F*(1, 27) = 1.53, *p* > 0.1, η_partial_^2^ = 0.05], or DHA [*F*(1, 20) = 2.75, *p* > 0.1, η_partial_^2^ = 0.12]. Finally, within-group paired *t*-tests showed that REP participants were the only group with a significant recall advantage for self-descriptive over not-self-descriptive words, for both negative words [*t*(22) = 2.23, *p* < 0.05, *d* = 0.95] and positive words [*t*(22) = 3.38, *p* < 0.01, *d* = 1.44; see also Figure 1C], and both these differences had large effect sizes. HA showed such self-descriptiveness advantage in recall only for negative words [*t*(27) = 2.63, *p* < 0.05, *d* = 1.01], but not positive words [*t*(27) = −0.19, *p* > 0.5, *d* = −0.07], DHA only for positive words [*t*(20) = 2.33, *p* < 0.05, *d* = 1.04], but not negative words [*t*(20) = −0.06, *p* > 0.5, *d* = −0.004]. The LA group showed neither significant self-descriptiveness advantage in recall [negative: *t*(26) = 0.22, *p* > 0.5, *d* = 0.09; positive: *t*(26) = 1.85, *p* < 0.1, *d*= 0.73], although approaching significance for positive words. Thus, the priming task evoked the strongest self-descriptiveness advantage in subsequent recall in REP, regardless of word valence.

### Priming

Word identification accuracy in the lexical decision task in experiment 3 was close to ceiling (proportions of 0.92 ± 0.05 of all items were correctly identified), did not differ between groups, and was therefore not analyzed further. Priming scores, subtracting response times to words from the encoding task from response times to new words, were analyzed, again as a function of valence (positive/negative), prior self-judgment (self-descriptive/not-self-descriptive), anxiety (high/low), and defensiveness (high/low). Controlling for the covariate “presence/absence of other participants during the experiment,” we found main effects of valence [*F*(1, 94) = 14.41, *p* < 0.001, η_partial_^2^ = 0.13] and self-judgment [*F*(1, 94) = 10.48, *p* < 0.01, η_partial_^2^ = 0.10]. Participants showed more priming for negative words [mean = 25.07 ms (SE = 3.2 ms)] than for positive words [mean = 9.57 ms (SE = 3.74 ms)] and more priming for self-descriptive words [mean = 22.54 ms (SE = 3.41 ms)] than for not-self-descriptive words [mean = 12.1 ms (SE = 2.87 ms)]. No further main effects or interactions were observed. The four-way interaction involving valence, self, anxiety, and defensiveness was far from significant [*F*(1, 94) = 0.4, *p* > 0.5, η_partial_^2^ = 0.004]. Thus, anxiety, defensiveness or their interaction did not influence priming performance at the delay.

## Discussion

Repressors were the only group without a recall advantage for negative words they had previously judged as self-descriptive compared to words they had not endorsed as self-descriptive in experiment 1. This replicates our previous results (Fujiwara et al., 2008) in an independent sample, despite the fact that in the current setting we used three times as many words, a more gradual 4-point self-judgment scale and only a self-judgment task (but no valence-judgment task). The gradual 4-point scale was used to allow more nuanced self-descriptiveness judgments than simple yes/no answers. By having less extreme options available we intended to allow participants to be more realistic in their self-judgments and intended to test more graded self-descriptiveness effects on memory. While we had to combine the 4-point answers into two categories (self-descriptive and not-self-descriptive) to avoid excessive loss of data, this type of answering during the encoding task critically differs from the simple two options during encoding in Fujiwara et al. (2008) and could have altered participants’ experience during encoding in the current experiment. This suggests that although the effect is small, it appears to be real. However, counter to our second hypothesis, this relatively lowered recall for negative self-descriptive information within the repressor group did not become more pronounced in free-recall after a 2-day delay in experiment 2. Note also that repressors consistently had the highest positivity-judgment ratio in each study, but only in study 1 did we see their relatively reduced recall.

We had good reasons to expect that self-descriptiveness may increase repressors’ recall biases over a delay. Hock and Krohne (Hock and Krohne, 2004; Krohne and Hock, 2008; Peters et al., 2012) found that repressors recalled threat-related information less than non-repressor groups only after a delay. The definition of repressive coping style by Weinberger (1990; Weinberger et al., 1979) states that individuals with a repressive coping style are not just sensitive to any threat but particularly to threat directed at their positive self-view. Various findings (e.g., in thought suppression or directed forgetting paradigms; Myers et al., 1998; Barnier et al., 2004; Myers and Derakshan, 2004) also point to a particular vulnerability of repressors to self-related threat. Our own previous study (Fujiwara et al., 2008) also found no immediate recall reductions in repressors for negative information that had simply been judged with regard to valence, but recall reductions occurred if encountered in a self-relevant encoding task.

There are several possible explanations why we did not observe stronger self-serving memory biases over the delay in repressors. First, the “threat” imposed by our task can reasonably be conceived as mild although the results of experiment 1 imply that even this mild threat was sufficient to show differential retrieval patterns against negative self-descriptive information only in repressors. Saunders et al. (2012) found that repressors are more prone to self-serving recall distortions in mnemic neglect paradigms than non-repressors, especially those with high anxiety levels. An important difference in our procedure compared to mnemic neglect paradigms is that here participants self-selected information as self-descriptive or not-self-descriptive rather than being given hypothetical self-view threatening feedback by the experimenter. Thus, it is possible that in our study, a motivation to retrieve information that had previously been deemed self-diagnostic superseded any potential self-protective biases in memory, and perhaps even more so after the delay. Secondly, our 2-day delay was (for practical reasons) shorter by a day than that in Krohne and Hock’s work. Thus, perhaps we did not detect self-serving recall biases in repressors after the 2-day delay simply due to a truncated wait time before such biases would emerge. However, we believe it is unlikely that this difference caused the difference in results. Repressors after 2 days started to show a self-descriptiveness advantage in free-recall, especially for positive words but also (non-significantly so) for negative words. It is difficult to imagine how repressors would first show a self-serving bias in immediate recall, which became eliminated after a 2-day delay, and which would then be reinstated or even exaggerated after 3 days.

Fading affect bias, a phenomenon describing faster decay of information associated with negative emotional experiences compared to positive emotions over time (Walker and Skowronski, 2009) may also have contributed to our results. After 2 days, any potential threat experienced by repressors immediately post-judgment, leading to immediate relative recall reductions for negative self-descriptive words, may have dissipated. To our knowledge, the effects of a test delay on the self-descriptiveness effect as tested in experiment 2 and 3 have not been reported previously. However, self-reference effects in memory (superior memory for information evaluated with reference to oneself, compared to information evaluated with reference to someone else) tend to become stronger over time (Symons and Johnson, 1997). Differences in self-judgments (self-descriptive/not-self-descriptive) explained 10% of the variance in free-recall at immediate test in experiment 1. They explained 17% at the delayed test in experiment 2, pointing to an increase of the self-descriptiveness effect in memory over a delay. Thus, self-descriptiveness effects, regardless of valence, may have counteracted any potential increase in self-serving memory biases in repressors over time. Results of experiment 3 can be interpreted in favor of this explanation as well. While cueing diminished self-descriptiveness effects in free-recall in all non-repressor groups, mainly due to increasing recall of not-self-descriptive information, repressors were the only group retaining the self-descriptiveness effect after cueing. Perhaps, repressors more so than any of the other groups, had been reminded of information they had previously endorsed as pertaining to themselves. They could have then used this information to produce the most self-consistent recall pattern rather than a positively biased one.

In this context, the assessment of coping styles using the BIDR-SDE subscale in our studies may have played a critical role. The BIDR-SDE subscale measures private, overconfident, egoistic self-deception (Paulhus, 1991; Paulhus and John, 1998) rather than outward-directed conformism to social norms as assessed with the Marlowe–Crowne Social Desirability Scale (MCSDS; Crowne and Marlowe, 1964), which is more commonly used in the repressive coping style literature following Weinberger et al. (1979). Repressors need to convince themselves and not just others of their own invulnerability against anxiety (Weinberger et al., 1979; Weinberger, 1990). Thus, the SDE part of the BIDR seemed better suited to assess the repressive coping style than the MCSDS (see also Weinberger and Davidson, 1994; Ashley and Holtgraves, 2003). However, individuals with high BIDR-SDE scores are also characterized by mental rigidity. For example, they perseverate in performing erroneous behavior in the face of failure (Peterson et al., 2003). It appears possible then that our high BIDR-SDE repressors may have reinterpreted initially self-devaluing information in a more positive light over the delay and therefore might have been well able to retrieve it, especially so when cued with an innocuous priming task. Therefore, despite the normative ratings used to select the words in this study (Anderson, 1968), it is possible that some of the negative (unlikable) words indeed represented desirable characteristics to repressors, particularly after some deliberation during the delay.

This interpretation does not run counter to Hock and Krohne’s findings (Hock and Krohne, 2004; Krohne and Hock, 2008; Peters et al., 2012). Rather it suggests that when assessing coping styles in different ways, memories for valenced materials and self-related materials decay differently over time depending on how repressive coping style is measured and whether self-involvement is present. Avoidance of (anxiety-induced) arousal underlies repressive coping in the MCI. Hence, arousal-inducing information may not be consolidated to the same extent in (MCI-)repressors than in groups of non-repressors, which could result in decreased memory for such information over time. Conversely, when using the BIDR-SDE to assess the defensiveness dimension of Weinberger’s conceptualization of repressive coping, information inconsistent with an over-positive self-view may become reinterpreted over time and seems to remain accessible.

Future extensions of the current studies would need to incorporate both valence- and self-descriptiveness judgments to test our suggestions. As such, one should assess whether repressors indeed judge originally self-descriptive negative information in a more positive way following a delay. Furthermore, the experiments involved either individual or group testing settings. Even though this seemed to have only minor influences on the results, it would be optimal to keep the testing environment more consistent in future studies, e.g., by only testing participants individually. An important limitation in this field in general is the relatively arbitrary separation of coping style groups. This can be remedied by using substantially larger samples and a continuous measure of repressive coping (as suggested by Mendolia, 2002). Another way to select repressive individuals might be to assess physiological reactivity (e.g., after a stress induction task) in conjunction with self-report, as done in some previous studies (e.g., Coifman et al., 2007). Repressors selected this way would show normal to high physiological reactivity in conjunction with an under-reporting of the stress experience, which is at the core of Weinberger’s characterization of the repressive coping style.

## Conclusion

In this set of studies, individuals with a repressive coping style showed selectively lowered immediate recall of negative self-descriptive information, but not after a 2-day delay. This result may seem to run counter to suggestions of repressors having an exaggerated bias against retrieving negative memories after a delay. However, we suggest that repressors, at least when assessed according to Weinberger’s classification scheme, may reinterpret initially negative self-relevant information in a more positive light after a delay, and therefore no longer experience the need to bias their recall at a delay.

## Conflict of Interest Statement

The authors declare that the research was conducted in the absence of any commercial or financial relationships that could be construed as a potential conflict of interest.

## Footnotes

^1^The grouping of individuals into one of the four coping styles (LA, HA, REP, DHA) using distinct cut-off scores based on the underlying sample is a somewhat arbitrary approach that has been criticized. Employing a combined score of defensiveness and anxiety (cf. Mendolia, 2002) can circumvent this issue and reanalysis of our results with such index score and hierarchical regression analyses did not qualitatively change our results, but lowered statistical power in some of the analyses. For reasons of clarity and to allow comparison with previous studies, we present our results in the more conventional categorical fashion.
